# Supplementary material for: Identification of Freezing-Responsive microRNAs and Their Targets in Chinese Jujube by Small RNA and Degradome Sequencing
Source: Int J Mol Sci. 2026 Sep 4;27(17):7912. doi: 10.3390/ijms27177912 (PMC13566890; doi:10.3390/ijms27177912)
Supplement: Supplementary file 1 [file ijms-27-07912-s001.zip › Supplementary Material S2/novel_miR_139.pdf]

Provisional ID	:	novel_miR_139
Score total	:	12108.3
Score for star read(s)	:	3.9
Score for read counts	:	12101.3
Score for mfe	:	1.5
Score for randfold	:	1.6
Score for cons. seed	:	
Total read count	:	23748
Mature read count	:	22886
Loop read count	:	18
Star read count	:	844

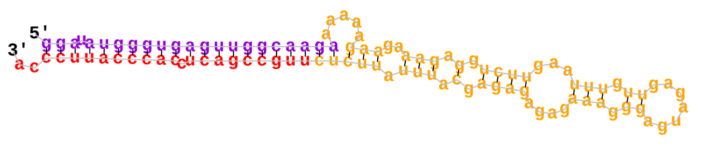

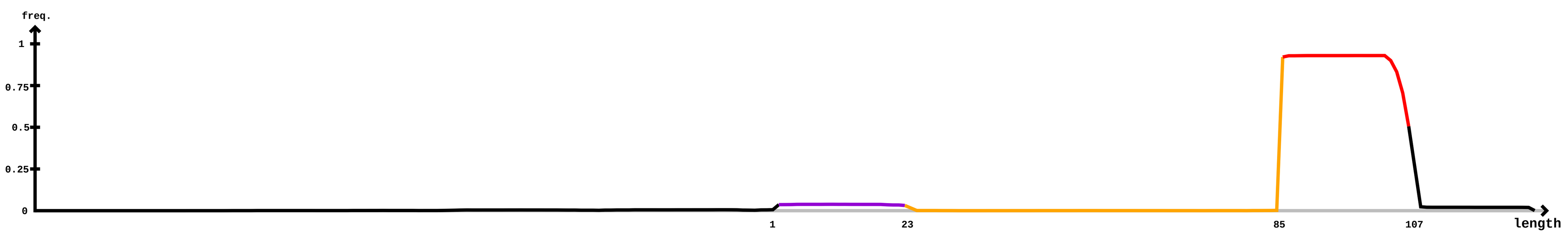

Star Mature

```
5'- augugcauaugcauauuaccaaaucuggaaccagagcuagccauagagggagagagagagaaagagagagauggacgagcuagagaagagggaaaaggaguaaagagcuuaaggaaguuuugggauaugggugaguuggcaagaaaaaagaagaaagaggucuugaauuuguugagaugagggaaagagagagagcauuuauucucuugccgacuccacccauucccaugauuuccuuuuguuccuccuu -3'   obs
    augugcauaugcauauuaccaaaucuggaaccagagcuagccauagagggagagagagagaaagagagagauggacgagcuagagaagagggaaaaggaguaaagagcuuaaggaaguuuugggauaugggugaguuggcaagaaaaaagaagaaagaggucuugaauuuguugagaugagggaaagagagagagcauuuauucucuugccgacuccacccauucccaugauuuccuuuuguuccuccuu        exp
    ((((((....))))))..(((..(((..........((..((......))..))............)))..)))....................((((((....((((..(((((((((.(((((.((((((((((((((((((.....(((..(((.(.((((...(((.((.......)).)))....)))).).))).))))))))))))))).))))))))))).)))))))))..))))))))))   reads   mm   sample
    ......................aucuggaaccagagcuagc.................................................................................................................................................................................................................   1   0   LT-3
    ........................cuggaaccagagcuagccauU.............................................................................................................................................................................................................   1   1   JCK-1
    ........................cuggaaccagagcuagccauU.............................................................................................................................................................................................................   1   1   JCK-3
    ..............................ccagagcuagccauagagggagagaga.................................................................................................................................................................................................   1   0   LT-2
    ...............................cagagcuagccauagagggagagaga.................................................................................................................................................................................................   5   0   LCK-3
    ...................................gcuagccauagagggagagagag................................................................................................................................................................................................   1   0   LCK-2
    ...................................gcuagccauagagggagagagagagaG............................................................................................................................................................................................   6   1   LCK-2
    ...................................gcuagccauagagggagagagagagaG............................................................................................................................................................................................   4   1   LCK-1
    ...............................................gggaAagagagagaaagagagag....................................................................................................................................................................................   1   1   LT-1
    ...................................................gagagGgagaaagagagaga...................................................................................................................................................................................   6   1   JT-2
    ...................................................gagagGgagaaagagagaga...................................................................................................................................................................................   3   1   JT-3
    ...................................................gagagagagaaagagagagaAg.................................................................................................................................................................................   1   1   LT-2
    ......................................................agagagaaagagagagaAgg................................................................................................................................................................................   1   1   LT-2
    ........................................................agagaaagagagagauggacgagcuagaga....................................................................................................................................................................   2   0   JT-2
    ........................................................agagaaagagagagauggacgagcuagaga....................................................................................................................................................................   3   0   JT-3
    .........................................................gagaGagagagagauggacgagc..........................................................................................................................................................................   2   1   LT-2
    ............................................................aGagagagagauggacgagcuagaga....................................................................................................................................................................   1   1   LT-1
    ...............................................................gagagagauggacgagcu.........................................................................................................................................................................   1   0   LT-1
    .................................................................gagagauggacgagcuag.......................................................................................................................................................................   2   0   JT-1
    ..................................................................agagauggacgagcuagagaaga.................................................................................................................................................................   4   0   JT-2
    ..................................................................agagauggacgagcuagagaaga.................................................................................................................................................................   7   0   JT-3
    ..................................................................agagauggacgagcuagagaagaggg..............................................................................................................................................................   1   0   JCK-2
    ...................................................................gagauggacgagcuagaga....................................................................................................................................................................   3   0   JCK-1
    ...................................................................gagauggacgagcuagagaa...................................................................................................................................................................   5   0   LCK-3
    ...................................................................gCgauggacgagcuagagaa...................................................................................................................................................................   1   1   LCK-3
    ...................................................................gagauggacgagcuagagaagaggga.............................................................................................................................................................   5   0   JCK-2
    ....................................................................agauggacgagcuagagaag..................................................................................................................................................................   3   0   LCK-3
    ....................................................................agauggacgagcuagagaagaggg..............................................................................................................................................................   11  0   JT-1
    ....................................................................agauggacgagcuagagaagagggaaaagg........................................................................................................................................................   2   0   JCK-1
    ....................................................................agauggacgagcuagagaagagggaaaagg........................................................................................................................................................   1   0   JCK-3
    .....................................................................gauggacgagcuagagaaga.................................................................................................................................................................   1   0   LT-3
    .....................................................................gauggacgagcuagagaaga.................................................................................................................................................................   3   0   JT-3
    .....................................................................gauggacgagcuagagaaga.................................................................................................................................................................   6   0   JT-2
```

Star Mature

augugcauaugcauauuaccaaaucuggaaccagagcuagccauagagggagagagagagaaagagagagauggacgagcuagagaagagggaaaaggaguaaagagcuuaaggaaguuuugggauaugggugaguuggcaagaaaaaagaagaaagaggucuugaauuuguugagaugagggaaagagagagagcauuuauucucuugccgacuccacccauucccaugauuuccuuuuguuccuccuu

gauggacgagcuagagaagaggg	8	0	LCK-3
gauggacgagcuagagaagaggg	3	0	JT-3
gauggacgagcuagagaagaggg	2	0	JT-2
gauggacgagcuagagaagagggaaaagg	1	0	JCK-2
auggacgagcuagagaaga	3	0	LT-2
auggacgagcuagagaaga	2	0	LT-3
auggacgagcuagagaagagggaaa	1	0	LT-1
auggacgagcuagagaagagggaaaagg	7	0	JT-1
auggacgagcuagagaagagggaaaagg	1	0	LT-1
uggacgagcuagagaagaggga	1	0	LT-3
uggacgagcuagagaagagggaaaagg	3	0	JT-1
gacgagcuagagaagagggaaaagg	3	0	LT-2
acgagcuagagaagagggaaaagga	1	0	LCK-3
cgagcuagagaagagggaaaagg	1	0	JCK-3
cgagcuagagaagagggaaaagg	1	0	JCK-1
agcuagagaagagggaaaagga	1	0	LT-3
cuagagaagagggaaaagg	1	0	LT-1
cuagagaagagggaaaaggag	1	0	LT-1
uagagaagagggaaaagg	1	0	LCK-3
agaagUgggaaaaggagua	1	1	LT-3
Uagggaaaaggaguaaagagc	1	1	LT-3
agggaaaaggaguaaagagc	1	0	LT-3
agggaaaaggaguaaagagc	3	0	LT-2
agggaaaaggaguaaagagc	1	0	LT-1
Aggaaaaggaguaaagagcuuaa	4	1	JCK-2
gggaaaaggaguaaagagcuuaagga	1	0	JCK-2
gaaaaggaguaaagagcuuaagg	1	0	JCK-3
aaaaggaguaaagagcuuaagg	1	0	LT-2
aaaaggaguaaagagcuuaagga	2	0	LT-2
aaaaggaguaaagagcuuaaggaag	2	0	LCK-3
aaaaggaguaaagagcuuaaggaag	4	0	LT-2
aaaggaguaaagagcuuaagU	2	1	JCK-3
aaaggaguaaagagcuuaagg	2	0	LT-3
aaaggaguaaagagcuuaagga	1	0	JCK-3
aaaggaguaaagagcuuaagga	1	0	JCK-2
aaaggaguaaagagcuuaagga	2	0	LT-2
aaaggaguaaagagcuuaagga	4	0	LCK-3
aaaggaguaaagagcuuaaggaag	4	0	JCK-1
aaaggaguaaagagcuuaaggaag	1	0	LT-1
aaaggaguaaagagcuuaaggaag	2	0	JCK-3
aaaggaguaaagagcuuaaggaagu	3	0	LT-2
aaaggaguaaagagcuuaaggaagu	12	0	LCK-3
aaaggaguaaagagcuuaaggaaguA	5	1	LCK-3
aaggaguaaagagcuuaagga	1	0	JCK-2
aggaguaaagagcuuaaggaC	4	1	LT-2
aggaguaaagagcuuaaggaa	2	0	LT-1
aggaguaaagagcuuaaggaag	1	0	LT-1
aggaguaaagagcuuaaggaaguu	3	0	LT-2
aggaguaaagagcuuaaggaaguu	2	0	LT-3
aggaguaaagagcuuaaggaaguuu	1	0	LT-2
aggaguaaagagcuuaaggaaguuu	1	0	JCK-3
aggaguaaagagcuuaaggaaguuA	1	1	LT-2
aggaguaaagagcuuaaggaaguuu	1	0	JCK-1
aggaguaaagagcuuaaggaaguuu	2	0	JCK-2
aguaaagagcuuaaggaa	13	0	JT-2
aguaaagagcuuaaggaa	15	0	JT-3
aguaaagagcuuaaggaaguu	1	0	JCK-3
aguaaagagcuuaaggaaguu	2	0	JCK-1
aguaaagagcuuaaggaaguu	2	0	LT-2
aguaaagagcuuaaggaaguu	1	0	LT-3
aguaaagagcuuaaggaaguuuug	1	0	LT-1
aguaaagagcuuaaggaaguuuug	2	0	LT-2
aguaaagagcuuaaggaaguuuug	6	0	LCK-3
agcuuaaggaaguuuugggauaugg	4	0	JCK-2
uuaaggaaguuuugggauaug	2	0	JCK-1
uuaaggaaguuuugggauaug	1	0	LT-3
uuaaggaaguuuugggauaug	1	0	JCK-3
uuaaggaaguuuugggauaugg	1	0	JCK-2
uuaaggaaguuuugggauaugggugagu	2	0	JCK-2
uuaaggaaguuuugggauaugggugagu	1	0	JCK-1

Star Mature

augugcauaugcauauuaccaaaucuggaaccagagcuagccauagagggagagagagagaaagagagagauggacgagcuagagaagagggaaaaggaguaaagagcuuaaggaaguuuugggauaugggugaguuggcaagaaaaaagaagaaagaggucuugaauuuguugagaugagggaaagagagagagcauuuauucucuugccgacuccacccauuccca ugauuuccuuuuguuccuccuu

aggaaguuuugggauaugggug	2	0	LT-1
ggaaguuuugggauaugggu	2	0	JT-3
ggaaguuuugggauaugggu	4	0	JT-2
ggaaguuuugggauauggguga	2	0	JCK-2
ggaaguuuugggauauggguga	1	0	JCK-3
ggaaguuuugggauauggguga	4	0	JCK-1
aguuuugggauaugggug	2	0	LT-2
uuuugggauaugggugaguuggca	4	0	LCK-3
uuuugggauaugggugaguuggca	1	0	LT-3
uugggauaugggugaguu	1	0	LT-1
uugggauaugggugaguugg	1	0	LT-1
uugggauaugggugaguuggc	11	0	JCK-2
uugggauaugggugaguuggc	9	0	JCK-1
uugggauaugggugaguuggc	3	0	LT-2
uugggauaugggugaguuggc	4	0	LT-3
uugggauaugggugaguuggc	6	0	JCK-3
uugggauaugggugaguuggca	3	0	JCK-1
uugggauaugggugaguuggca	3	0	JCK-2
uugggauaugggugaguuggca	8	0	JCK-3
uugggauaugggugaguuggcaag	1	0	JT-3
uugggauaugggugaguuggcaag	2	0	JCK-1
uugggauaugggugaguuggcaag	1	0	JCK-3
uugggauaugggugaguuggcaag	1	0	JCK-2
uugggauaugggugaguuggcaaga	1	0	LT-1
uugggauaugggugaguuggcaaga	4	0	JCK-3
uugggauaugggugaguuggcaaga	1	0	JCK-1
uugggauaugggugaguuggcaaga	4	0	JCK-2
ugggauaugggugaguuggc	2	0	JCK-1
ugggauaugggugaguuggc	2	0	JCK-3
ugggauaugggugaguuggc	1	0	LT-1
ugggauaugggugaguuggca	2	0	JCK-2
ugggauaugggugaguuggca	2	0	LT-1
ugggauaugggugaguuggcaag	1	0	JCK-2
ugggauaugggugaguuggcaaAa	1	1	LT-3
Cggauaugggugaguuggcaaga	3	1	JT-3
Cggauaugggugaguuggcaaga	1	1	LT-3
Cggauaugggugaguuggcaaga	1	1	JCK-1
Cggauaugggugaguuggcaaga	3	1	LT-2
Cggauaugggugaguuggcaaga	1	1	LT-1
Cggauaugggugaguuggcaaga	8	1	JT-2
Cggauaugggugaguuggcaaga	4	1	JCK-3
Cggauaugggugaguuggcaaga	6	1	JT-1
Cggauaugggugaguuggcaaga	2	1	JCK-2
ggauaugggugaguuggc	2	0	LT-3
ggauaugggugaguuggc	1	0	JCK-1
ggauaugggugaguuggc	2	0	JCK-3
ggauaugggugaguuggc	6	0	LT-2
ggauaugggugaguuggca	1	0	LT-3
ggauaugggugaguuggca	4	0	JCK-3
ggauaugggugaguuggca	1	0	JCK-2
ggauaugggugaguuggca	1	0	LT-1
ggauaugggugaguuggcaC	2	1	LT-3
ggauaugggugaguuggcaag	13	0	JCK-3
ggauaugggugaguuggcaag	10	0	JCK-1
ggauaugggugaguuggcaag	4	0	LT-2
Agauaugggugaguuggcaag	1	1	JCK-2
ggauaugggugaguuggcaag	3	0	LT-1
ggauaugggugaguuggcaag	10	0	LT-3
ggauaugggugaguuggcaag	5	0	LCK-3
ggauaugggugaguuggcaag	12	0	JCK-2
ggauaugggugaguuggcaaga	73	0	JCK-3
ggCuaugggugaguuggcaaga	1	1	JCK-1
ggauaugggugaguuggcaaga	62	0	LT-2
ggauaugggugaguuggcaGga	1	1	LCK-3
ggauCugggugaguuggcaaga	1	1	JT-1
ggauaugggugCguuggcaaga	1	1	JCK-1
ggauaugggugaguuggcaaga	121	0	JCK-2
gCauaugggugaguuggcaaga	1	1	JCK-3
gAauaugggugaguuggcaaga	1	1	JT-1
ggauaugggugaguuggcaaga	51	0	LT-3

Star Mature

augugcauaugcauauuaccaaaucuggaaccagagcuagccauagagggagagagagagaaagagagagauggacgagcuagagaagagggaaaaggaguaaagagcuuaaggaaguuuugggauaugggugaguuggcaagaaaaaagaagaaagaggucuugaauuuguugagaugagggaaagagagagagcauuuauucucuugccgacuccacccauucccaugauuuccuuuuguuccuccuu

ggauaugggugaguuggcaaga	44	0	LT-1
ggauaugggugaguuggcaaga	23	0	JT-2
ggauaugggugaguuggcaaga	26	0	JT-3
ggaCaugggugaguuggcaaga	1	1	LCK-3
ggCuaugggugaguuggcaaga	3	1	LT-2
ggauaugggugaguuggcaaga	90	0	LCK-3
ggauaugggugaguuggcaagC	1	1	JT-1
ggauauUggugaguuggcaaga	1	1	JT-2
Agauaugggugaguuggcaaga	1	1	LT-3
ggauaugggugaguuggcaagN	1	1	LT-2
ggauaugggugaguuggcaaga	88	0	JT-1
ggauaugggugaguuggcaaga	81	0	JCK-1
ggauaugggugaguuggcaagaa	1	0	JCK-3
ggauaugggugaguuggcaagaa	1	0	JCK-2
ggauaugggugaguuggcaagaU	2	1	JCK-2
gauaugggugaguuggcaag	4	0	JCK-2
auaugggugaguuggcaag	4	0	LCK-3
auaugggugaguuggcaaga	1	0	JCK-2
auaugggugaguuggcaaga	1	0	LT-1
auaugggugaguuggcaagaa	1	0	JCK-2
uaugggugaguuggcaaga	1	0	LT-3
uaugggugaguuggcaaga	6	0	JT-2
uaugggugaguuggcaaga	21	0	JT-3
augggugaguuggcaaga	1	0	JCK-1
augggugaguuggcaaga	1	0	JCK-2
augggugaguuggcaaga	1	0	JCK-3
gugaguuggcaagaaaaaa	7	0	LCK-3
gugaguuggcaagaaaaaagaa	3	0	JT-2
gugaguuggcaagaaaaaagaa	2	0	JT-3
gugaguuggcaagaaaaaagaa	1	0	JCK-2
ugaguuggcaagaaaaaag	2	0	LT-2
Uguuggcaagaaaaaaga	3	1	JCK-1
aguuggcaagaaaaaagaa	3	0	LT-3
agaagaaagaggucuugaau	1	0	JCK-3
gaaagaggucuugaauuugu	2	0	LCK-3
aggucuugaauuuguugag	1	0	LT-3
cuugaauuuguugagaugagg	2	0	JCK-1
cuugaauuuguugagaugagggaaagag	1	0	LT-2
ugaauuuguugagaugaggg	1	0	LT-3
aauuuguugagaugaggga	1	0	LT-1
auuuguugagaugaggga	1	0	LT-1
auuuguugagaugagggaaagagaga	3	0	LT-2
uuguugagaugagggaaaA	1	1	LT-1
ugagaugagggaaagagagagagcauuu	3	0	LCK-3
aaagagagagagcauuuGuuc	1	1	LT-1
agagcauuuauucucuugccgacuccacA	1	1	JCK-3
gcauuuGuucucuugccgacuccacccau	2	1	JCK-3
cauuuGuucucuugccgacucc	1	1	JCK-2
cauuuGuucucuugccgacucc	1	1	JCK-1
uucucuugccgacuccacc	1	0	LT-1
uucucuugccgacuccacccauu	6	0	LCK-3
uucucuugccgacuccacccauuccc	1	0	JT-3
uucucuugccgacuccacccauuccc	5	0	JT-2
uucucuugccgacuccacccauucccUu	1	1	JCK-3
cucuugccgacuccacccauu	1	0	LT-2
cucuugccgacuccacccauuc	2	0	JCK-2
cucuugccgacuccacccauucc	1	0	JCK-2
ucuugccgacuccacccauuc	2	0	JCK-2
ucuugccgacuccacccauucc	1	0	JCK-3
ucuugccgacuccacccauucc	1	0	JCK-1
ucuugccgacuccacccauucc	2	0	JCK-2
cuugccgacuccacccauucc	1	0	LT-3
cuugccgacuccacccauuccc	1	0	LT-1
cuugccgacuccacccauuccc	2	0	JCK-3
cuugccgacuccacccauuccc	2	0	LT-3
cuugccgacuccacccauuccc	2	0	JCK-2
cuugccgacuccacccauuccca	1	0	JCK-1
cuugccgacuccacccauuccca	5	0	JCK-2
cuugccgacuccacccauuccca	3	0	LT-1
cuugccgacuccacccauuccca	2	0	JCK-3

Star / Mature

augugcauaugcauauuaccaaaucuggaaccagagcuagccauagagggagagagagagaaagagagagauggacgagcuagagaagagggaaaaggaguaaagagcuuaaggaaguuuugggauaugggugaguuggcaagaaaaaagaagaaagaggucuugaauuuguugagaugagggaaagagagagagcauuuauucucuugccgacuccacccauucccaugauuuccuuuuguuccuccuu

uugccgacuccacccauA	1	1	LT-3
uugccgacuccacccauu	45	0	LCK-3
uugccgacuccacccauu	111	0	JCK-2
uugccgacuccacccauA	4	1	JCK-2
uugccgacuccacccauA	5	1	JCK-3
uugccgacuccCcccauu	1	1	LT-3
uugccgacuccaAccauu	1	1	JCK-3
uugccgacuccacccauu	19	0	JT-1
uugccgacuccacccauu	31	0	LCK-1
uugccgacuccacccauu	138	0	LT-3
uugccgacuccacccauA	4	1	JCK-1
uugccgacuccacccauA	19	1	LCK-3
uugccgacuccacccauu	5	0	JT-2
uugccgacuccacccauu	4	0	JT-3
uugccgacuccacccauu	51	0	JCK-1
uugccgacuccacccaCu	1	1	JCK-2
uugccgacuccacccauu	76	0	LT-2
uugccgacuccacccauu	71	0	JCK-3
uugccgacuccacccauA	5	1	LT-2
uugccgacuccacccauA	3	1	LT-1
uugccgacuccacccauu	34	0	LCK-2
uugccgacuccacccauu	88	0	LT-1
uugccgacuUcacccauu	4	1	LCK-3
uugccAacuccacccauu	1	1	JT-1
uugccgacuccacccauC	2	1	LT-1
uugccgacuccacccauuU	38	1	JCK-1
uugccgacuccacccauuU	65	1	JCK-2
uugccgacuccacccaAuc	1	1	LT-3
uugccgacuccacccauuc	167	0	LT-2
uugccgCcuccacccauuc	1	1	LT-1
uugccgacuccacccauuU	21	1	JT-2
uugccgacuccacccauuU	6	1	LCK-2
uugccgacuccacccauuA	4	1	LCK-3
uugccgacuccacccauuU	51	1	LT-3
uugccgacuccacccauuU	15	1	JT-3
uugccAacuccacccauuc	1	1	LT-1
uugccgacuccacccauuc	171	0	LCK-3
uugccgacuccCcccauuc	1	1	LT-1
uugccgacuccacccauuA	3	1	JCK-2
uugccgacuccacccauuU	36	1	JT-1
uugccgacuccacccauuc	52	0	JT-2
uugccgacuccacccauuU	5	1	LCK-1
uugccgacuccacccauuA	3	1	JCK-1
uugccgacuccacccauuc	82	0	JCK-3
uugccgacuccacccauuU	49	1	LT-2
uugccgacuccacccaAuc	1	1	LT-2
uugccgacuccaccNauuc	1	1	JT-3
uugccAacuccacccauuc	2	1	LT-2
uugccgacuccacccauuc	150	0	JCK-2
uugccgacuccacccauuA	6	1	LT-3
uNgccgacuccacccauuc	1	1	JCK-2
uugccgacuccacccauuA	1	1	JCK-3
uugccgacuccacccauuA	5	1	LT-1
uugUcgacuccacccauuc	1	1	LCK-3
uugccgacuccacccauuc	234	0	LT-3
uugccgacuccacccauuc	84	0	JT-3
uugccgacuccacccauuc	44	0	JT-1
uugccgacuccacccauuc	147	0	LT-1
uugccgCcuccacccauuc	1	1	LT-3
uugccgacuccacccauuU	78	1	LCK-3
uuAccgacuccacccauuc	1	1	LT-3
uugccgacuccacccauuU	35	1	JCK-3
uugccgCcuccacccauuc	1	1	LCK-3
uugccgacuccacccauuA	1	1	LT-2
uugccgacuccacccauuc	56	0	JCK-1
uugccgacuccacccauuU	32	1	LT-1
uugccgacuccCcccauucc	1	1	LT-1
uugccgacuccacccauucc	118	0	JT-1
uugccgacuccacccCuucc	1	1	LT-2
uugccgacuccacccauucc	116	0	LCK-1

```
                                                                                                                          Star                                                                                Mature
augugcauaugcauauuaccaaaucuggaaccagagcuagccauagagggagagagagagaaagagagagauggacgagcuagagaagagggaaaaggaguaaagagcuuaaggaaguuuugggauaugggugaguuggcaagaaaaaagaagaaagaggucuugaauuuguugagaugagggaaagagagagagcauuuauucucuugccgacuccacccauucccaugauuuccuuuuguuccuccuu
..............................................................................................................................................................................................................uugUcgacuccacccauucc........................  1    1   JCK-3
..............................................................................................................................................................................................................uugccgacuccacccauucU........................  26   1   LT-1
..............................................................................................................................................................................................................uugccgacuccCcccauucc........................  2    1   JCK-2
..............................................................................................................................................................................................................uugccgaUuccacccauucc........................  2    1   LCK-2
..............................................................................................................................................................................................................uugccgCcuccacccauucc........................  2    1   JCK-2
..............................................................................................................................................................................................................uugccgaAuccacccauucc........................  1    1   JCK-3
..............................................................................................................................................................................................................uugccUacuccacccauucc........................  4    1   JCK-3
..............................................................................................................................................................................................................uugccgacuccacccauucU........................  25   1   JCK-2
..............................................................................................................................................................................................................uugccgacuccacccauuUc........................  4    1   JT-2
..............................................................................................................................................................................................................uugccgacuccacccauucU........................  22   1   LT-2
..............................................................................................................................................................................................................uugccgacuccacccauucU........................  20   1   LCK-3
..............................................................................................................................................................................................................uugccgacuccUcccauucc........................  1    1   JCK-2
..............................................................................................................................................................................................................uugccgacuccacccauuUc........................  6    1   JCK-1
..............................................................................................................................................................................................................uugccgacuccacccauucA........................  9    1   LT-2
..............................................................................................................................................................................................................uugccgaAuccacccauucc........................  1    1   JCK-2
..............................................................................................................................................................................................................uugccgaAuccacccauucc........................  1    1   LT-1
..............................................................................................................................................................................................................uugccgCcuccacccauucc........................  1    1   JT-1
..............................................................................................................................................................................................................uugccgacuccacccauucc........................  481  0   LT-3
..............................................................................................................................................................................................................uugccgacuccaccUauucc........................  2    1   LT-3
..............................................................................................................................................................................................................uugccgacuccacccauucA........................  9    1   LT-3
..............................................................................................................................................................................................................uugccgacuccacccauuUc........................  10   1   LT-3
..............................................................................................................................................................................................................uugccgacuccacccauucc........................  336  0   LT-1
..............................................................................................................................................................................................................uugccgacuccaccUauucc........................  1    1   JCK-3
..............................................................................................................................................................................................................uugccgacucUacccauucc........................  1    1   LT-3
..............................................................................................................................................................................................................uugccgacuccacccaCucc........................  1    1   JT-1
..............................................................................................................................................................................................................uugccgacuccacccauucc........................  134  0   LCK-2
..............................................................................................................................................................................................................uugccgacuccacccauucA........................  4    1   JCK-3
..............................................................................................................................................................................................................uugccgacuccacccauuUc........................  11   1   JT-1
..............................................................................................................................................................................................................uugccgacuccacccauucA........................  3    1   LT-1
..............................................................................................................................................................................................................uugccgacuccacccauucU........................  2    1   JT-2
..............................................................................................................................................................................................................uugccgaAuccacccauucc........................  1    1   LT-2
..............................................................................................................................................................................................................uugccgacuccacccauuUc........................  11   1   LT-1
..............................................................................................................................................................................................................uugccgacuccacccauucA........................  5    1   LCK-3
..............................................................................................................................................................................................................uugccgacuccacccauucc........................  282  0   LCK-3
..............................................................................................................................................................................................................uugccgacuccacccauucU........................  14   1   JCK-1
..............................................................................................................................................................................................................uugccgacuccacccauuUc........................  13   1   LT-2
..............................................................................................................................................................................................................uugccgacuccacccauucc........................  221  0   JCK-1
..............................................................................................................................................................................................................uugccgaUuccacccauucc........................  1    1   JCK-2
..............................................................................................................................................................................................................uugccgacuccacccauucc........................  77   0   JT-3
..............................................................................................................................................................................................................uugccgacuccNcccauucc........................  1    1   JT-2
..............................................................................................................................................................................................................uugccgacuccacccauAcc........................  1    1   LT-1
..............................................................................................................................................................................................................uugccgacuccacccauucc........................  370  0   JCK-2
..............................................................................................................................................................................................................uugccgacuccacccauuUc........................  5    1   JT-3
..............................................................................................................................................................................................................uugccgacuccCcccauucc........................  1    1   LT-3
..............................................................................................................................................................................................................uugccgacuccacccauuUc........................  5    1   JCK-2
..............................................................................................................................................................................................................uugccgacuccacccauucA........................  2    1   JCK-1
..............................................................................................................................................................................................................uugccgacuccacccauucc........................  303  0   LT-2
..............................................................................................................................................................................................................uugccgacuccacccauucc........................  300  0   JCK-3
..............................................................................................................................................................................................................uugccgacuccacccauuUc........................  3    1   JCK-3
..............................................................................................................................................................................................................uugccgaUuccacccauucc........................  1    1   LT-3
..............................................................................................................................................................................................................uugccAacuccacccauucc........................  1    1   JCK-2
..............................................................................................................................................................................................................uugccgacuccCcccauucc........................  1    1   JCK-3
..............................................................................................................................................................................................................uugccgacuccacccauucA........................  10   1   JT-1
..............................................................................................................................................................................................................uugccgacuccNcccauucc........................  1    1   JCK-1
..............................................................................................................................................................................................................uugccgacuccacccauucU........................  4    1   JT-3
..............................................................................................................................................................................................................uugccgacuccacccauucU........................  7    1   JCK-3
..............................................................................................................................................................................................................uugccgacuAcacccauucc........................  1    1   JCK-3
..............................................................................................................................................................................................................uugccgacCccacccauucc........................  1    1   LCK-1
..............................................................................................................................................................................................................uugccgacuccacccauucU........................  30   1   LT-3
..............................................................................................................................................................................................................uugccgacuccacccauucc........................  59   0   JT-2
..............................................................................................................................................................................................................uugccgaAuccacccauucc........................  1    1   JT-2
..............................................................................................................................................................................................................uugcAgacuccacccauucc........................  1    1   JCK-2
..............................................................................................................................................................................................................uugccgacuccacccauucA........................  6    1   JCK-2
..............................................................................................................................................................................................................uugUcgacuccacccauucc........................  1    1   JCK-1
..............................................................................................................................................................................................................uugccgacuccCcccauucc........................  1    1   JT-1
..............................................................................................................................................................................................................uugccgacuccCcccauuccc.......................  1    1   JCK-3
..............................................................................................................................................................................................................uugccgacuUcacccauuccc.......................  1    1   LT-1
..............................................................................................................................................................................................................uugccgacuccacccauuccc.......................  59   0   JT-2
..............................................................................................................................................................................................................uugccgacuAcacccauuccc.......................  1    1   LT-3
..............................................................................................................................................................................................................uugccgacuccacccauuccA.......................  1    1   LT-1
```

Star Mature

```
augugcauaugcauauuaccaaaucuggaaccagagcuagccauagagggagagagagagaaagagagagauggacgagcuagagaagagggaaaaggaguaaagagcuuaaggaaguuuugggauaugggugaguuggcaagaaaaaagaagaaagaggucuugaauuuguugagaugagggaaagagagagagcauuuauucucuugccgacuccacccauucccaugauuuccuuuuguuccuccuu
..............................................................................................................................................................................................................uugccgacuUcacccauuccc.......................   1     1   JCK-3
..............................................................................................................................................................................................................uugccgacuccacccauuccA.......................   2     1   JCK-3
..............................................................................................................................................................................................................uugccgacuccacccauucUc.......................   2     1   JCK-3
..............................................................................................................................................................................................................uugccgaAuccacccauuccc.......................   1     1   LCK-2
..............................................................................................................................................................................................................uugccgacuccacccauuccA.......................   6     1   LCK-3
..............................................................................................................................................................................................................uugccgacuccacccauuccc.......................   820   0   LT-3
..............................................................................................................................................................................................................uugccgacuccacccauuccc.......................   590   0   JCK-1
..............................................................................................................................................................................................................uugUcgacuccacccauuccc.......................   1     1   JCK-2
..............................................................................................................................................................................................................uugccgCcuccacccauuccc.......................   1     1   JT-2
..............................................................................................................................................................................................................uugccgacuccacccauuccc.......................   976   0   JCK-2
..............................................................................................................................................................................................................uugccgacuccacccauuccc.......................   728   0   JCK-3
..............................................................................................................................................................................................................uugccgacuccacccauuccA.......................   1     1   JCK-1
..............................................................................................................................................................................................................uugccgacuccacccauuccc.......................   248   0   LCK-3
..............................................................................................................................................................................................................uugccgacuccacccauuccA.......................   2     1   LT-3
..............................................................................................................................................................................................................uugccgacuccaUccauuccc.......................   2     1   LT-3
..............................................................................................................................................................................................................uugccgCcuccacccauuccc.......................   1     1   LT-3
..............................................................................................................................................................................................................uugccgacuccacccauuccc.......................   90    0   LCK-2
..............................................................................................................................................................................................................uugccgacuccacccauGccc.......................   1     1   JCK-1
..............................................................................................................................................................................................................uuNccgacuccacccauuccc.......................   2     1   LT-2
..............................................................................................................................................................................................................uugccUacuccacccauuccc.......................   1     1   JCK-3
..............................................................................................................................................................................................................uugccgacuccacccauCccc.......................   1     1   JCK-1
..............................................................................................................................................................................................................uugccgCcuccacccauuccc.......................   2     1   JCK-2
..............................................................................................................................................................................................................uugccgaAuccacccauuccc.......................   1     1   LT-3
..............................................................................................................................................................................................................uugUcgacuccacccauuccc.......................   2     1   JCK-1
..............................................................................................................................................................................................................uugccgacuccacccauuccN.......................   2     1   JCK-1
..............................................................................................................................................................................................................uugccgacuccaAccauuccc.......................   1     1   LT-3
..............................................................................................................................................................................................................uugccgacuccacccCuuccc.......................   3     1   LT-3
..............................................................................................................................................................................................................Cugccgacuccacccauuccc.......................   1     1   LT-2
..............................................................................................................................................................................................................uugccgacuccGcccauuccc.......................   2     1   JCK-1
..............................................................................................................................................................................................................uugccgacuccacUcauuccc.......................   1     1   LT-1
..............................................................................................................................................................................................................uugccgacuccaAccauuccc.......................   1     1   JCK-1
..............................................................................................................................................................................................................uugccgacuccacccauuccc.......................   581   0   LT-1
..............................................................................................................................................................................................................uugccgacuccCcccauuccc.......................   1     1   LT-1
..............................................................................................................................................................................................................uugccgacuUcacccauuccc.......................   1     1   JCK-2
..............................................................................................................................................................................................................uugccgacuccCcccauuccc.......................   1     1   JCK-2
..............................................................................................................................................................................................................uugccgacuccacccauuccU.......................   5     1   JCK-2
..............................................................................................................................................................................................................uugccgacuccacccauuccU.......................   5     1   JCK-3
..............................................................................................................................................................................................................uugccgacuccacccauucUc.......................   1     1   JCK-1
..............................................................................................................................................................................................................Augccgacuccacccauuccc.......................   1     1   LT-2
..............................................................................................................................................................................................................uugccgaAuccacccauuccc.......................   1     1   LT-2
..............................................................................................................................................................................................................uugccgacuccacccauucUc.......................   2     1   LT-2
..............................................................................................................................................................................................................uugccgaAuccacccauuccc.......................   1     1   JCK-3
..............................................................................................................................................................................................................uugccgaAuccacccauuccc.......................   1     1   LT-1
..............................................................................................................................................................................................................uugccgacuUcacccauuccc.......................   1     1   JCK-1
..............................................................................................................................................................................................................uuAccgacuccacccauuccc.......................   1     1   JCK-2
..............................................................................................................................................................................................................uugccgacuccacccauuccA.......................   1     1   JCK-2
..............................................................................................................................................................................................................uugccgacuccCcccauuccc.......................   2     1   LT-3
..............................................................................................................................................................................................................uugccgacuccacccauuccc.......................   84    0   LCK-1
..............................................................................................................................................................................................................uugccgacuccaccUauuccc.......................   1     1   JCK-2
..............................................................................................................................................................................................................uNgccgacuccacccauuccc.......................   3     1   JCK-2
..............................................................................................................................................................................................................uugccgCcuccacccauuccc.......................   1     1   LT-2
..............................................................................................................................................................................................................uugccgacuccacccauuccU.......................   1     1   LCK-3
..............................................................................................................................................................................................................uNgccgacuccacccauuccc.......................   2     1   JCK-1
..............................................................................................................................................................................................................uugccgacuccaccUauuccc.......................   2     1   JCK-3
..............................................................................................................................................................................................................uugccgacuccacccauuccA.......................   4     1   LT-2
..............................................................................................................................................................................................................uugccgacuccacccauuccc.......................   378   0   LT-2
..............................................................................................................................................................................................................uugccgacuccacccCuuccc.......................   3     1   JCK-3
..............................................................................................................................................................................................................uugccgacuccacccauuccU.......................   14    1   LT-3
..............................................................................................................................................................................................................uugccgaAuccacccauuccc.......................   2     1   JCK-1
..............................................................................................................................................................................................................uugccgacuccaAccauuccc.......................   1     1   LCK-2
..............................................................................................................................................................................................................uugccgacuccacccauuUcc.......................   2     1   JCK-3
..............................................................................................................................................................................................................uugccgacuccaAccauuccc.......................   2     1   LT-2
..............................................................................................................................................................................................................uugccgacuccacccauuccU.......................   4     1   JT-1
..............................................................................................................................................................................................................uugccgacuccacccCuuccc.......................   1     1   LCK-3
..............................................................................................................................................................................................................uugccAacuccacccauuccc.......................   2     1   LT-3
..............................................................................................................................................................................................................uugNcgacuccacccauuccc.......................   1     1   JCK-1
..............................................................................................................................................................................................................uugccgacuccaAccauuccc.......................   1     1   LCK-3
..............................................................................................................................................................................................................uugccgacuccacccauucUc.......................   2     1   JCK-2
..............................................................................................................................................................................................................uugccgacucAacccauuccc.......................   1     1   LT-1
..............................................................................................................................................................................................................uugccgacuccaAccauuccc.......................   1     1   LT-1
```

Star Mature

augugcauaugcauauuaccaaaucuggaaccagagcuagccauagagggagagagagagaaagagagagauggacgagcuagagaagagggaaaaggaguaaagagcuuaaggaaguuuugggauaugggugaguuggcaagaaaaaagaagaaagaggucuugaauuuguugagaugagggaaagagagagagcauuuauucucuugccgacuccacccauucccaugauuuccuuuuguuccuccuu

uugccgacuccacccauuccc	75	0	JT-3
uugccgacuccaAccauuccc	1	1	JCK-3
uugccgacuccacccauuccc	114	0	JT-1
uugccgacuccacccauuccU	11	1	LT-2
uugccgaAuccacccauuccc	5	1	JCK-2
uugccgacuccacccauucUc	2	1	LT-3
uugccgacuccaAccauuccca	3	1	LT-3
uugccgacuccacccauuccNa	3	1	JCK-1
Cugccgacuccacccauuccca	1	1	JCK-2
uugccgacuccacccauucccN	1	1	JT-2
uugccgacuccacccauuUcca	4	1	JCK-2
uugccgacucAacccauuccca	2	1	JCK-1
uugccgacuccacccCuuccca	6	1	JCK-2
uugccgacuccacccauuccca	259	0	LCK-3
uugccgacuccacccauGccca	3	1	JCK-1
uCgccgacuccacccauuccca	1	1	JCK-1
uugccgacuccacccauuccca	69	0	JT-2
uugccgacuccCcccauuccca	1	1	LCK-3
uugccgacuccacccauucUca	4	1	JCK-2
uugccgacuccacccauucccN	1	1	JCK-1
uugccgacuccacccauuccca	75	0	LCK-1
uugccgacuccacccauuAcca	1	1	JCK-3
uugccgacuccacccauucccG	2	1	LT-3
uugccgacuccCcccauuccca	5	1	JCK-2
uugccgCcuccacccauuccca	7	1	JCK-2
uugccgacuccaUccauuccca	2	1	JCK-3
uugccgaAuccacccauuccca	3	1	LT-1
uugccgaAuccacccauuccca	4	1	LT-3
uugccgacuccacccauuccca	848	0	LT-1
uugccgaUuccacccauuccca	1	1	LT-1
uugccgaAuccacccauuccca	25	1	JCK-2
uugccgacuccacccauucccG	1	1	LT-1
uugccgacuccacccauuAcca	1	1	JCK-2
uuNccgacuccacccauuccca	3	1	LT-2
uugccgacuccacccCuuccca	2	1	LT-3
uugccgacuccacccauucUca	1	1	JCK-3
uugccgacuccacccauGccca	2	1	JCK-3
uuNccgacuccacccauuccca	1	1	LT-3
uugccgacuccacccauucccC	2	1	JCK-3
uugccgaUuccacccauuccca	1	1	LT-2
uugccgacuccUcccauuccca	4	1	JCK-1
uugccgacuccGcccauuccca	1	1	JCK-1
uugccgCcuccacccauuccca	2	1	JCK-3
uugcUgacuccacccauuccca	1	1	JCK-3
uugccgCcuccacccauuccca	2	1	LT-3
uugccgacucAacccauuccca	1	1	LCK-2
uuUccgacuccacccauuccca	1	1	JCK-2
uugccgacuccacccauucccG	2	1	JCK-2
uugccgacuccacccauuccca	1059	0	LT-3
uugUcgacuccacccauuccca	1	1	LT-1
uugccgacuccacccauucccG	3	1	JCK-3
uugccgacuccacccauuUcca	2	1	JCK-1
uugccgacuccacccauuccca	453	0	LT-2
uugGcgacuccacccauuccca	1	1	JCK-3
uugcAgacuccacccauuccca	1	1	JCK-2
uugccgacuccacccauucccU	2	1	LT-1
uugccgacuccacccauuccUa	4	1	LT-3
uugccgacuccacccauucccG	1	1	JCK-1
uugccgacuccUcccauuccca	1	1	JCK-3
uugccgacuccaAccauuccca	1	1	JCK-3
uugccgacuccacccauucUca	1	1	JCK-1
uugccgacucAacccauuccca	1	1	LT-1
uugccgacuccCcccauuccca	2	1	LT-2
uugccgacuccacccauucccU	6	1	JCK-1
uugccgacuccacccauCccca	1	1	JCK-2
uugccgacucUacccauuccca	2	1	JCK-3
uugccgaAuccacccauuccca	3	1	LT-2
uugGcgacuccacccauuccca	2	1	JCK-1
uugccAacuccacccauuccca	1	1	JCK-1
uugccgacuccacccCuuccca	1	1	JCK-1

```
Star                                                                            Mature
augugcauaugcauauuaccaaaucuggaaccagagcuagccauagagggagagagagagaaagagagagauggacgagcuagagaagagggaaaaggaguaaagagcuuaaggaaguuuugggauaugggugaguuggcaagaaaaaagaagaaagaggucuugaauuuguugagaugagggaaagagagagagcauuuauucucuugccgacuccacccauucccaugauuuccuuuuguuccuccuu
..............................................................................................................................................................................................................uugccgacuccacccauCccca......................  1     1  JCK-3
..............................................................................................................................................................................................................uugccgacuccacccauuccca......................  2863  0  JCK-3
..............................................................................................................................................................................................................uugccgacuccGcccauuccca......................  1     1  LT-2
..............................................................................................................................................................................................................uugccgacuccacccauCccca......................  2     1  LT-3
..............................................................................................................................................................................................................uugccgacuUcacccauuccca......................  1     1  JCK-2
..............................................................................................................................................................................................................uugccgacuccacccauucccC......................  4     1  JCK-1
..............................................................................................................................................................................................................uugccgCcuccacccauuccca......................  1     1  LT-2
..............................................................................................................................................................................................................uugccgacuccaAccauuccca......................  4     1  JCK-2
..............................................................................................................................................................................................................uugccNacuccacccauuccca......................  1     1  JCK-3
..............................................................................................................................................................................................................uugccgacuccCcccauuccca......................  2     1  LT-1
..............................................................................................................................................................................................................uugccgacucUacccauuccca......................  5     1  JCK-2
..............................................................................................................................................................................................................uugccgaAuccacccauuccca......................  12    1  JCK-3
..............................................................................................................................................................................................................uugccgacuccacccauuccca......................  3808  0  JCK-2
..............................................................................................................................................................................................................uugccgacuUcacccauuccca......................  3     1  JCK-3
..............................................................................................................................................................................................................uugccgacuccCcccauuccca......................  1     1  JT-2
..............................................................................................................................................................................................................uugccgCcuccacccauuccca......................  1     1  JT-1
..............................................................................................................................................................................................................uugccgacucGacccauuccca......................  1     1  JCK-1
..............................................................................................................................................................................................................uugccgacuGcacccauuccca......................  1     1  JCK-3
..............................................................................................................................................................................................................uugUcgacuccacccauuccca......................  1     1  LT-3
..............................................................................................................................................................................................................uugccgacuccCcccauuccca......................  1     1  JCK-3
..............................................................................................................................................................................................................uugccgacuccacccauuGcca......................  1     1  JCK-1
..............................................................................................................................................................................................................uugUcgacuccacccauuccca......................  1     1  JCK-1
..............................................................................................................................................................................................................uugccgacuccacccauCccca......................  1     1  JCK-1
..............................................................................................................................................................................................................uugccgacuccNcccauuccca......................  2     1  JCK-1
..............................................................................................................................................................................................................uugccgacucAacccauuccca......................  1     1  JCK-2
..............................................................................................................................................................................................................uugccAacuccacccauuccca......................  2     1  JCK-3
..............................................................................................................................................................................................................uugccgaUuccacccauuccca......................  1     1  JCK-2
..............................................................................................................................................................................................................uugccgacuccaUccauuccca......................  1     1  JCK-2
..............................................................................................................................................................................................................uugccgacuccacccGuuccca......................  1     1  JCK-2
..............................................................................................................................................................................................................uugccgacuccacccauucccU......................  3     1  JCK-2
..............................................................................................................................................................................................................uugccgacuccaccUauuccca......................  1     1  LT-3
..............................................................................................................................................................................................................uugccgacuccacccauucNca......................  3     1  JCK-1
..............................................................................................................................................................................................................uugccgacuUcacccauuccca......................  1     1  JCK-1
..............................................................................................................................................................................................................uNgccgacuccacccauuccca......................  6     1  JCK-1
..............................................................................................................................................................................................................uugccgaAuccacccauuccca......................  12    1  JCK-1
..............................................................................................................................................................................................................uugccUacuccacccauuccca......................  1     1  JCK-3
..............................................................................................................................................................................................................uugccgacucAacccauuccca......................  3     1  JCK-3
..............................................................................................................................................................................................................uNgccgacuccacccauuccca......................  5     1  JCK-2
..............................................................................................................................................................................................................uugccgacuccacccauuUcca......................  4     1  JCK-3
..............................................................................................................................................................................................................uugccgacuccaAccauuccca......................  1     1  JCK-1
..............................................................................................................................................................................................................uugccgacuccacccaCuccca......................  1     1  JCK-2
..............................................................................................................................................................................................................uugUcgacuccacccauuccca......................  3     1  JCK-2
..............................................................................................................................................................................................................uugNcgacuccacccauuccca......................  3     1  JCK-1
..............................................................................................................................................................................................................uugccgacuccacccauucccN......................  1     1  LT-2
..............................................................................................................................................................................................................uugccgacuccacccauuccca......................  76    0  LCK-2
..............................................................................................................................................................................................................uugccgacuccacccauucccU......................  2     1  JCK-3
..............................................................................................................................................................................................................uugccgacuccacccauuccca......................  92    0  JT-3
..............................................................................................................................................................................................................uugccgacuccaAccauuccca......................  1     1  LT-2
..............................................................................................................................................................................................................uugccgacuccacccauuNcca......................  1     1  JCK-1
..............................................................................................................................................................................................................uugccgacuccacccauucccC......................  6     1  JCK-2
..............................................................................................................................................................................................................uugccgacuccacccauuccca......................  63    0  JT-1
..............................................................................................................................................................................................................uugccgaUuccacccauuccca......................  1     1  JCK-1
..............................................................................................................................................................................................................uCgccgacuccacccauuccca......................  2     1  JCK-3
..............................................................................................................................................................................................................uugccgacuccacccauuccca......................  2210  0  JCK-1
..............................................................................................................................................................................................................uugUcgacuccacccauuccca......................  2     1  JCK-3
..............................................................................................................................................................................................................uugccgacuccacccauuccUa......................  10    1  JT-1
..............................................................................................................................................................................................................Cugccgacuccacccauuccca......................  1     1  JCK-1
..............................................................................................................................................................................................................uugccgacucGacccauuccca......................  1     1  JCK-2
..............................................................................................................................................................................................................uugccgacuccacccauuccUa......................  1     1  LT-1
..............................................................................................................................................................................................................uugccgCcuccacccauuccca......................  1     1  JCK-1
..............................................................................................................................................................................................................uugccgacuccacccauucccUu.....................  2     1  JCK-2
..............................................................................................................................................................................................................uugccgacuccacccauucccaA.....................  6     1  LT-1
..............................................................................................................................................................................................................uugccgacuccacccauucccau.....................  8     0  JCK-3
..............................................................................................................................................................................................................uugccgacuccacccauucccaA.....................  7     1  LCK-3
..............................................................................................................................................................................................................uugccgacuccacccauucccaA.....................  9     1  JCK-3
..............................................................................................................................................................................................................uugccgacuccacccauucccaA.....................  16    1  JCK-2
..............................................................................................................................................................................................................uugccgacuccacccauucccUu.....................  3     1  JCK-1
..............................................................................................................................................................................................................uugccgacuccacccauucccaG.....................  2     1  LT-1
..............................................................................................................................................................................................................uugccgacuccacccauucccaA.....................  17    1  JCK-1
..............................................................................................................................................................................................................uugccgacuccacccauucccau.....................  6     0  JCK-1
```

```
                                                                                                                                                    Star                                                              Mature
augugcauaugcauauuaccaaaucuggaaccagagcuagccauagagggagagagagagaaagagagagauggacgagcuagagaagagggaaaaggaguaaagagcuuaaggaaguuuugggauaugggugaguuggcaagaaaaaagaagaaagaggucuugaauuuguugagaugagggaaagagagagagcauuuauucucuugccgacuccacccauucccaugauuuccuuuuguuccuccuu
..............................................................................................................................................................................................................uugccgacuccacccauucccau.....................   1   0   LT-3
..............................................................................................................................................................................................................uugccgacuccacccauuccAau.....................   2   1   LT-3
..............................................................................................................................................................................................................uugccgacuccacccauucccaC.....................   1   1   JCK-2
..............................................................................................................................................................................................................uugccgacuccacccauucccau.....................   9   0   JCK-2
..............................................................................................................................................................................................................uugccgacuccacccauucccaG.....................   1   1   LT-3
..............................................................................................................................................................................................................uugccgacuccacccauucccaC.....................   4   1   LT-1
..............................................................................................................................................................................................................uugccgacuccacccauucccaGg....................   1   1   LT-3
..............................................................................................................................................................................................................uugccgacuccacccauucccauC....................   1   1   JCK-1
..............................................................................................................................................................................................................uugccgacuccacccauucccauU....................   6   1   JCK-2
..............................................................................................................................................................................................................uugccgacuccacccauucccauC....................   3   1   LT-2
..............................................................................................................................................................................................................uugccgacuccacccauucccauU....................   1   1   JCK-3
..............................................................................................................................................................................................................uugccgacuccacccauucccaug....................   1   0   LT-1
..............................................................................................................................................................................................................uugccgacuccacccauucccaug....................   7   0   JCK-1
..............................................................................................................................................................................................................uugccgacuccacccauucccauU....................   1   1   LT-1
..............................................................................................................................................................................................................uugccgacuccacccauucccaug....................   5   0   JCK-3
..............................................................................................................................................................................................................uugccgacuccacccauucccauU....................   2   1   LT-3
..............................................................................................................................................................................................................uugccgacuccacccauucccaAg....................   1   1   LT-1
..............................................................................................................................................................................................................uugccgacuccacccauucccaug....................   11  0   JCK-2
..............................................................................................................................................................................................................uugccgacuccacccauucccauU....................   3   1   JCK-1
..............................................................................................................................................................................................................uugccgacuccacccauucccauA....................   1   1   LT-1
..............................................................................................................................................................................................................uugccgacuccacccauucccaug....................   2   0   LT-2
..............................................................................................................................................................................................................uugccgacuccacccauucccaugU...................   1   1   JCK-3
...............................................................................................................................................................................................................ugccgacuccacccauuc.........................   2   0   JCK-1
...............................................................................................................................................................................................................ugccgacuccacccauuc.........................   13  0   JT-1
...............................................................................................................................................................................................................ugccgacuccacccauuc.........................   1   0   LT-1
...............................................................................................................................................................................................................ugccgacuccacccauucc........................   1   0   JCK-3
...............................................................................................................................................................................................................ugccgacuccacccauucc........................   2   0   LT-2
...............................................................................................................................................................................................................ugccgacuccacccauucc........................   1   0   LT-3
...............................................................................................................................................................................................................ugccgacuccaAccauuccc.......................   1   1   LT-3
...............................................................................................................................................................................................................ugccgacuccacccauuccc.......................   3   0   LT-3
...............................................................................................................................................................................................................ugccgacuccacccauuccc.......................   20  0   LCK-2
...............................................................................................................................................................................................................ugccgacuccacccauuccc.......................   2   0   JCK-3
...............................................................................................................................................................................................................ugccgacuccacccauuccc.......................   23  0   LCK-1
...............................................................................................................................................................................................................ugccgacuccacccauuccc.......................   2   0   JCK-1
...............................................................................................................................................................................................................ugccgacuccacccauuccc.......................   4   0   LCK-3
...............................................................................................................................................................................................................ugccgacuccacccauuccca......................   9   0   LT-2
...............................................................................................................................................................................................................ugccgacuccacccauuccca......................   9   0   LCK-3
...............................................................................................................................................................................................................ugccgacuccacccauuccca......................   19  0   LT-1
...............................................................................................................................................................................................................ugccgaAuccacccauuccca......................   1   1   LT-2
...............................................................................................................................................................................................................ugccgacuccacccauuccca......................   26  0   LT-3
...............................................................................................................................................................................................................ugccgacuccacccauuccca......................   14  0   JCK-3
...............................................................................................................................................................................................................ugccgacuccacccauuccca......................   14  0   JCK-2
...............................................................................................................................................................................................................ugccgacuccacccauuccca......................   11  0   JCK-1
...............................................................................................................................................................................................................ugccgacuUcacccauuccca......................   1   1   JCK-3
...............................................................................................................................................................................................................ugccgacuUcacccauuccca......................   1   1   JCK-2
...............................................................................................................................................................................................................uUccgacuccacccauuccca......................   1   1   LT-2
...............................................................................................................................................................................................................ugccgacuccacccauucccauC....................   3   1   JCK-2
.................................................................................................................................................................................................................ccgacuccacccauucccaug....................   2   0   JCK-1
.................................................................................................................................................................................................................ccgacuccacccauucccaug....................   4   0   JCK-2
.................................................................................................................................................................................................................ccgacuccacccauucccaug....................   1   0   LT-3
.................................................................................................................................................................................................................ccgacuccacccauucccaug....................   1   0   LT-1
.................................................................................................................................................................................................................ccgacuccacccauucccauga...................   2   0   JCK-2
.................................................................................................................................................................................................................ccgacuccacccauucccauga...................   1   0   JCK-1
..................................................................................................................................................................................................................cgacuccacccauuccca......................   2   0   LT-1
..................................................................................................................................................................................................................cgacuccacccauucccaug....................   2   0   LT-3
..................................................................................................................................................................................................................cgacuccacccauucccaug....................   3   0   JCK-2
..................................................................................................................................................................................................................cgacuccacccauucccaug....................   2   0   JCK-1
..................................................................................................................................................................................................................cgacuccacccauucccaug....................   2   0   JCK-3
..................................................................................................................................................................................................................cgacuccacccauucccauga...................   1   0   JCK-1
..................................................................................................................................................................................................................cgacuccacccauucccaugauuuccuu............   2   0   JCK-1
........................................................................................................................................................................................................................cacccauucccaugauuucc..............   3   0   LT-3
..........................................................................................................................................................................................................................cccauucccaugauuuccuu............   5   0   LT-2
............................................................................................................................................................................................................................cauucccaugauuuccuuuugu........   1   0   JCK-1
..............................................................................................................................................................................................................................uucccaugauuuccuuuuguuccucc..   1   0   LT-1
.................................................................................................................................................................................................................................ccaugauuuccuuuuguuccucc..   1   0   LT-3
...................................................................................................................................................................................................................................augauuuccuuuuguuccucc..   3   0   JCK-1
...................................................................................................................................................................................................................................augauuuccuuuuguuccucc..   2   0   LT-2
...................................................................................................................................................................................................................................augauuuccuuuuguuccucc..   1   0   JCK-2
....................................................................................................................................................................................................................................ugauuuccuuuuguuccu....   1   0   JCK-2
....................................................................................................................................................................................................................................ugauuuccuuuuguuccu....   1   0   LT-3
```

```
                                                                                                                                              Star                                                                                    Mature
augugcauaugcauauuaccaaaucuggaaccagagcuagccauagagggagagagagagaaagagagagauggacgagcuagagaagagggaaaaggaguaaagagcuuaaggaaguuuugggauaugggugaguuggcaagaaaaaagaagaaagaggucuugaauuuguugagaugagggaaagagagagagcauuuauucucuugccgacuccacccauucccaugauuuccuuuuguuccuccuu
....................................................................................................................................................................................................................................ugauuuccuuuuguuccuc...   3   0   JCK-1
....................................................................................................................................................................................................................................ugauuuccuuuuguuccuc...   1   0   JCK-2
....................................................................................................................................................................................................................................ugauuuccuuuuguuccuc...   5   0   LT-2
....................................................................................................................................................................................................................................ugauuuccuuuuguuccuc...   1   0   LT-1
....................................................................................................................................................................................................................................ugauuuccuuuuguuccuc...   4   0   LT-3
....................................................................................................................................................................................................................................ugauuuccuCuuguuccucc..   1   1   JCK-3
....................................................................................................................................................................................................................................ugauuuccuuuuguuccucc..   52  0   JCK-1
....................................................................................................................................................................................................................................ugauuuccuuuuguuccucc..   64  0   LCK-3
....................................................................................................................................................................................................................................ugauuCccuuuuguuccucc..   1   1   LT-2
....................................................................................................................................................................................................................................ugauuuccuuuuguuccucc..   40  0   LT-1
....................................................................................................................................................................................................................................ugaCuuccuuuuguuccucc..   1   1   JCK-2
....................................................................................................................................................................................................................................ugCuuuccuuuuguuccucc..   1   1   LT-2
....................................................................................................................................................................................................................................ugauuuccuuuuguuccucc..   59  0   LT-3
....................................................................................................................................................................................................................................ugauuuccuuuuguuccucc..   4   0   JT-2
....................................................................................................................................................................................................................................ugauuuccuuuuguuccucc..   8   0   JT-3
....................................................................................................................................................................................................................................ugauuuccuuuuguuccucc..   60  0   LT-2
....................................................................................................................................................................................................................................ugauuuccuuuuguuccucA..   1   1   LT-2
....................................................................................................................................................................................................................................ugGuuuccuuuuguuccucc..   1   1   JCK-2
....................................................................................................................................................................................................................................ugauuuccuuuuguuccucc..   63  0   JCK-3
....................................................................................................................................................................................................................................ugCuuuccuuuuguuccucc..   1   1   LT-3
....................................................................................................................................................................................................................................ugauuuccuuuuguuccucc..   92  0   JCK-2
....................................................................................................................................................................................................................................ugauuuccuuuuguuccuccu.   3   0   JCK-1
....................................................................................................................................................................................................................................ugauuuccuuuuguuccuccu.   3   0   JCK-3
....................................................................................................................................................................................................................................ugauuuccuuuuguuccuccG.   1   1   JCK-2
....................................................................................................................................................................................................................................ugauuuccuuuuguuccuccA.   7   1   LCK-3
....................................................................................................................................................................................................................................ugauuuccuuuuguuccuccA.   1   1   JCK-1
....................................................................................................................................................................................................................................ugauuuccuuuuguuccuccu.   5   0   JCK-2
```
